# Detecting Anomalies of Satellite Power Subsystem via Stage-Training Denoising Autoencoders

**DOI:** 10.3390/s19143216

**Published:** 2019-07-22

**Authors:** Weihua Jin, Bo Sun, Zhidong Li, Shijie Zhang, Zhonggui Chen

**Affiliations:** 1 Research Center of Satellite Technology, Harbin Institute of Technology, Harbin 150080, China; 2 Beijing Institute of Spacecraft System Engineering, Beijing 100094, China

**Keywords:** satellite power subsystem, anomaly detection, stage-training denoising autoencoder

## Abstract

Satellite telemetry data contains satellite status information, and ground-monitoring personnel need to promptly detect satellite anomalies from these data. This paper takes the satellite power subsystem as an example and presents a reliable anomaly detection method. Due to the lack of abnormal data, the autoencoder is a powerful method for unsupervised anomaly detection. This study proposes a novel stage-training denoising autoencoder (ST-DAE) that trains the features, in stages. This novel method has better reconstruction capabilities in comparison to common autoencoders, sparse autoencoders, and denoising autoencoders. Meanwhile, a cluster-based anomaly threshold determination method is proposed. In this study, specific methods were designed to evaluate the autoencoder performance in three perspectives. Experiments were carried out on real satellite telemetry data, and the results showed that the proposed ST-DAE generally outperformed the autoencoders, in comparison.

## 1. Introduction

Anomalies might occur in all subsystems of satellites in orbit. Anomalies in some subsystems are fatal to satellites, such as guidance, navigation, control, and power subsystems. A lot of work has been done to ensure the normal operation of satellites. This paper focuses on the anomaly detection of power subsystems. As an important part of the satellite, the satellite power subsystem is responsible for all power supply in a satellite. The performance of the system directly affects the working status of other systems and affects the life of the satellite [1]. Hence, detecting anomalous behavior of satellite power subsystem is an important task to prevent failures that result in the unwanted outcome or even cause damage to the system and developing a complete, accurate, and reliable anomaly detecting system is the goal of every national satellite expert.

The satellite manufacturing industry intensively utilizes numerous sensors to seamlessly monitor the temperature and electrical properties of machines. The recorded sensor data can be examined to distinguish normal from unexpected behavioral patterns [2]. On-orbit satellites generate large amounts of telemetry data every day, which are large-scale and multivariate time-series data. Moreover, many instances of normal behavior are available, whereas the number of anomalous samples is limited. Hence, the challenge in anomaly detection lies in modeling the normal patterns and detecting previously unseen patterns that might hint at machine failure. In the past, this was often accomplished by engineers with sufficient knowledge of the domain. However, this is often expensive in terms of time. Therefore, the anomaly detection mothed of satellite power subsystem must have the capability to efficiently complete this unsupervised task [3].

Anomaly detection on multivariate time-series data is of great importance in both fundamental machine learning research and industrial applications [4]. The main challenges of anomaly detection in multivariate time-series data can be summarized as follows [2,5,6]:

1. Definition of anomalous behavior—The boundary between normal and anomalous behavior is often not precise, so it is very difficult to define a normal region that encompasses every possible normal behavior. Thus, an anomalous observation that lies close to the boundary can actually be normal, and vice-versa.
2. Irrelevant features—A high proportion of irrelevant features effectively creates noise in the input data, which masks the true anomalies. The challenge is to choose a subspace of the data that highlights the relevant attributes.
3. Bias of Scores—Scores based on $L_{p}$ norms are biased toward high-dimensional subspaces, if they are not normalized appropriately. In particular, distances in different dimensionality (and thus distances measured in different subspaces) are not directly comparable.

Fruitful methods for anomaly detection have been made in the last several years [7,8]. These methods can be grouped into two categories—(1) traditional machine learning techniques and (2) deep learning. Traditional machine learning include techniques such as, support vector machines (SVMs) [6,9,10,11], Principal Component Analysis (PCA) [3,12,13], k-means algorithm [14], and Hidden Markov Models (HMM) [15]. The development of deep learning provides new methods for anomaly detection, such as Long Short-Term Memory Networks (LSTM) [16,17] and the autoencoder [2,4,18]. SVMs, especially the One-Class Support Vector Machines (1SVMs) are a popular technique for unsupervised anomaly detection. However, training SVMs is memory-intensive and time-intensive. SVMs are non-parametric learning models, whose complexity grows greatly with the number of records [6]. For the large-scale and multivariate time-series satellite telemetry data, inherent shortcomings exist in SVMs. PCA projects high-dimensional time-series into a low-dimensional sequence. However, during the transformation process, information losses of multivariate time-series become inevitable [15]. HMM also has the limitation of intensive computing, especially in case of fuzzy integral-based detectors. Moreover, HMM needs to manually specify some initial values of degrees of importance. LSTM is an artificial Recurrent Neural Network (RNN) architecture that has recently been shown to be very effective for anomaly detection in standard time-series test data. However, if the time-series data are not stationary, the algorithm performance decreases. An autoencoder adopts the neural network architecture to perform unsupervised learning, which is trained to reconstruct instances of normal time-series with the target time-series being the input time-series itself. Then, the reconstruction error is used to compute the likelihood of anomaly, at that point [16]. However, due to the interaction between different features, the autoencoder often extract meaningless or wrong high-level features that cause the autoencoder model to become unreliable. Extraction of the correct features cannot be guaranteed during the autoencoder training, in most cases.

The main objective of this study was to make the autoencoder model more reliable and robust by incorporating expert knowledge into the process of training. Another objective of this study was to develop an anomaly detector based on the autoencoder and demonstrate that the proposed method can effectively detect anomalous behavior of a satellite power subsystem. In this study, we proposed stage-training denoising autoencoders (ST-DAE) to accomplish these two objectives. The first step was to do enough data exploration with expert knowledge and discover the features of the data. Then, the features were trained through stage-training denoising autoencoders (ST-DAE) until the model became reliable and robust. After this, we could reconstruct the test time-series and compute the likelihood of anomaly, based on the reconstruction errors. Finally, the anomaly threshold could be determined through a cluster-based method and the anomalies could be discovered on the basis of the threshold. The paper is organized as follows. Section 2 introduces the basics of the satellite power subsystem and autoencoder. Section 3 describes the research methodology, mainly including the autoencoder training strategy and anomaly evaluation method. The experiment results are shown and discussed in Section 4. Conclusions and future work are discussed in Section 5.

## 2. Background

### 2.1. Satellite Power Subsystem Description

**Figure 1:**
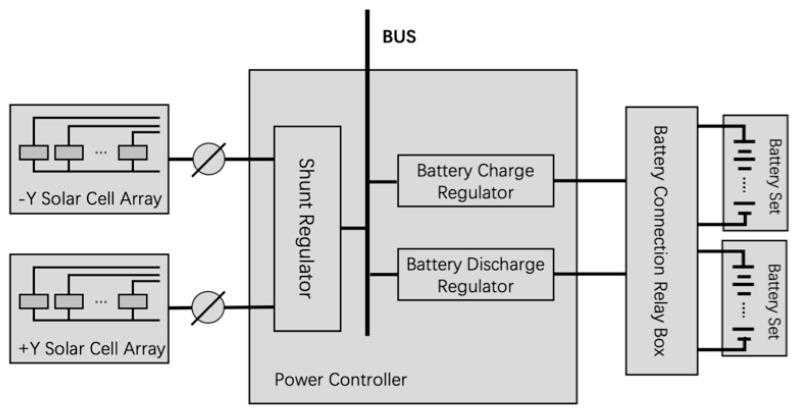
Diagram of the satellite power subsystem.

The satellite power subsystem converts different forms of energy to achieve energy distribution and scheduling, thereby completing the on-orbit task. As shown in Figure 1, the satellite power subsystem mainly includes the solar cell array, battery set, shunt regulator (SR), battery discharge regulator (BDR), battery charge regulator (BCR), and battery connection relay box [3].

The main electric energy comes from solar cells when the satellite runs in space. When the satellite enters the illumination area, the solar panels are photoelectrically converted. Parallel switching regulators control the electrical energy of the solar cells connected to the bus. When the satellite enters the shadow area from the illumination area, the voltage of the solar cell decreases gradually as the illumination decreases. All the parallel switch regulators that are not connected to the bus successively transfer the power of the solar cell to the circuit, until all solar cells are connected. When the illumination intensity decreases to a certain extent and the bus voltage cannot be maintained by the parallel switch regulator, the battery discharge regulator starts to work. At this time, the solar cell and the battery set supply power to the load at the same time, but the power supply capacity of the solar cell decreases, and finally, the power supply stops. Satellites in the shadows are powered entirely by the battery set. Although the light gradually increases, the discharge regulator still works. When the light meets certain conditions, the discharge regulator withdraws from work, and then the charging regulator starts to work. At this time, the solar cell supplies electrical energy to the load, on the one hand, and charges the battery set through the charging regulator on the other hand. When the illumination is further strengthened (when the satellite operates in the illumination area), the output current of the charging regulator reaches the maximum value and becomes the constant current charge of the battery set.

### 2.2. The Autoencoder

**Figure 2:**
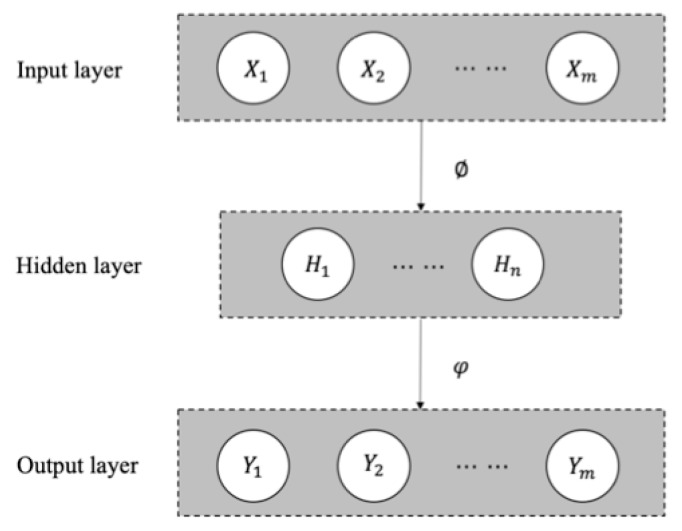
The autoencoder architecture (n < m).

An autoencoder was first introduced to address the problem of “backpropagation without a teacher”, in the 1980s [19]. An autoencoder is a type of artificial neural network used to learn efficient data coding as an unsupervised technique, in which we leverage neural networks for the task of representation learning, typically for dimensionality reduction. As shown in Figure 2, we can take an unlabeled dataset and frame it as a supervised learning problem by predicting the target value Y as close as possible to its original input X. This network can be trained by minimizing the reconstruction error, ℒ(X,Y), which measures the differences between our original input and the output reconstruction [20]. The autoencoder always consists of two parts, the encoder and the decoder, which can be defined as transitions ∅ and φ. The ∅=S(WX+b) defines the transition X→H, where W and b are the matrices of weights and biases, respectively, and S represents the activation functions. Similarly, the $\varphi =S\left(W^{\prime }H\;+\;b^{\prime }\right)$ defines the transition H→Y. Therefore, the loss can be defined as $argmin{\left\|X-Y\right\|}^{2}$. An autoencoder is capable of discovering structure hidden in data, in order to develop a compressed representation *H* of the input. 

For this purpose, many different variants of the general autoencoder architecture, such as denoising autoencoders (DAE) [21] and sparse autoencoders (SAE) [22], were developed to ensure the compressed representation represents meaningful attributes of the original data input. Denoising autoencoders are trained to reconstruct a clean “repaired” input from a corrupted version $\tilde{X}$, which is done by adding noises to input data X by means of a stochastic mapping $\tilde{X}\sim q_{D}\left(\tilde{X}|X\right)$. In this case, a good representation can be obtained robustly from a corrupted input $\tilde{X}$ which would be useful for recovering the corresponding clean input X. There are three recommended ways to add noises into input data [23]—(1) additive isotropic Gaussian noise, (2) masking noise, which is a fraction of the input values that are randomly selected and forced to be 0, (3) salt-and-pepper noise, which is a fraction of the input values that are randomly selected and set to their minimum or maximum possible value (typically 0 or 1). Sparse autoencoders are the ones whose numbers of hidden units are large (perhaps even greater than the number of input pixels), however, we can still discover an interesting structure, by imposing a sparsity constraint on the hidden units, then the autoencoder would still discover interesting structure in the data, even if the number of hidden units is large. ‘Sparse’ can be understood as follows—a neuron is considered as “active” if its output value is close to 1, or as “inactive” if its output value is close to 0. We would like to constrain the neurons to be inactive most of the time [24].

## 3. Research Methodology

### 3.1. Data Exploration and Preprocessing

Data exploration is the initial and important approach to understand the characteristics of the telemetry data. In order to establish an effective anomaly detection model, enough data exploration work should be done. As numerous sensors are set on the satellite to monitor the status of the satellite, it also causes a redundancy in telemetry data. Therefore, the first step is to select meaningful features based on expert knowledge. The second step is to traverse each feature to check the completeness and correctness of the data. Missing data are common occurrences, which can have a significant effect on the conclusions that can be drawn from the data. There are several methods to handle missing data [25]—imputation [26], pairwise deletion [27], and sensitivity analysis [28]. Finally, for the time-series, we need to plot every feature on the x-axis (time) and find out the changing rule of each feature over time, and the relationships between features. Then, the features can be grouped on the basis of observations and analysis, as well as domain knowledge. All of the above steps can create a clear mental model and understanding of the data in the mind of the domain experts, and define basic metadata (statistics, structure, relationships) for the data set, which can be used in further analysis [29].

### 3.2. Stage-Training Denoising Autoencoders

**Figure 3:**
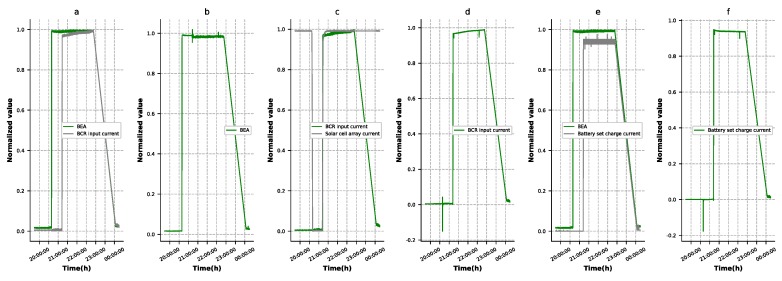
High reconstruction error caused by the interaction between features. (**a**) The BEA signal value and BCR input current. (**b**) The reconstruction result of the BEA signal value. (**c**) The BCR input current and solar cell array current. (**d**) The reconstruction result of the BCR input current. (**e**) The BEA signal value and battery set charge current. (**f**) The reconstruction result of the battery set charge current.

An autoencoder can encode and decode data distributions. Ideally, an autoencoder would have a smaller test error and would learn efficient latent features of the data set when the autoencoder is in training [30]. However, it is often observed that autoencoders end up obtaining small test errors but are not able to learn efficient latent vector of the data. When the telemetry data is trained with a autoencoder, we find the following problems. As shown in Figure 3a, the battery error amplifier (BEA) signal value rises after the BCR input current rises, then their values are partially similar. After the autoencoder model is trained, we find that the reconstructed BEA signal value has large reconstruction errors, where the BCR input current rises as shown in Figure 3b. Solar cell array current and BCR input current, as shown in Figure 3c,d, face this problem, while the BEA signal value and battery set charge current, as shown in Figure 3e,f, have the same issue. The reason for this is that the autoencoders have a powerful fitting ability, but the latent high-level features learned from the data set may be deviated due to the interaction between the features.

In this study, stage-training denoising autoencoders (ST-DAE) were developed to isolate this adverse effect, as described above. The first step was to group all features based on the results of data exploration and the features in each group had the same characteristics and did not affect each other during the training. Then, we determined the structure of the autoencoder, and selected one group of features to keep the values unchanged and change the values of the remaining features into a constant. In this case, the autoencoder mode was easy to train and extract latent high-level features from among the selected ones, as most features had the same value. In the next stage, the autoencoder model was first initialized with the weight of the autoencoder model trained in the previous stage. Then, we selected another group of features among the remaining ones to change back to the original value and trained these data into this autoencoder model. This operation was looped until all the groups of features were trained. 

Four sets of control experiments that were constructed with three types of autoencoders and two training methods were set up, as shown in Table 1. ‘AE’, ‘SAE’, and ‘DAE’ represent common, sparse, and denoising autoencoders, respectively. The autoencoder is denoted by the prefix ‘ST-’ if the stage-training method was used, else it is denoted as ‘T-’.

### 3.3. Performance Evaluation

#### 3.1.1. Computing the Anomaly Scores and Anomaly Threshold

There are two ways to calculate the anomaly scores. The anomaly score for $t_{i}$ can be calculated by the root mean squared error $e=\sqrt{\frac{\sum_{i=1}^{n}{\left(x_{i}-x_{i}^{'}\right)}^{2}}{s}}$, where s is the count of features [18]. The error e can be directly used as an anomaly score. Another method is to treat the root mean squared errors as observations, which can be used to estimate the parameters μ and ∑ of a Normal distribution N(μ,∑), using the Maximum Likelihood Estimation (MLE). Then, the anomaly score $a_{i}=\;{\left(e_{i}-\mu \right)}^{T}\sum^{-1}\left(e_{i}-\mu \right)$ for each point at $t_{i}$ [16]. 

Due to the lack of anomaly labels, it can be difficult to define the anomaly threshold. Two general methods are usually used [18]—(1) specify the threshold based on domain expertise; (2) specify the threshold based on common assumptions of anomalies, e.g., 5%–10% of the data are anomalous candidates. In this study, a novel method is presented to define the anomaly threshold. Considering the anomaly scores, they can be divided into a normal value and an abnormal value, so the anomaly scores could be clustered into the normal cluster and an anomalous cluster. In this way, we could automatically get the anomaly threshold. K-means clustering [31] was used in our study.

#### 3.1.2. Model Evaluation

It is difficult to evaluate the performance of unsupervised anomalous detection due to the lack of anomaly labels [18]. In this study, the proposed methods were evaluated from three perspectives. First, the well-developed autoencoders needed to be capable of efficiently limiting the false alarm rate. False alarms occur when autoencoders do not learn all characteristics of the normal data. To check the false alarm rate in the normal dataset is a good method of evaluating the model. The false alarm rate is expected to be lower for autoencoders with better reconstruction abilities. These four autoencoder models, as described in Table 1, could test the ability to limit false alarms on the test data sets. The most important step is to determine anomaly threshold in this progress. In this study, we used a common autoencoder (T-AE) as the baseline method and the anomaly threshold could be determined on the basis of the reconstruction results generated by the ‘T-AE’. After getting the reconstruction result, the anomaly scores could be obtained by calculating the root mean squared error of the input data and the reconstruction data. Then, the anomaly scores could be clustered into two clusters by the K-means clustering method and the minimum anomaly score of the abnormal cluster was the anomaly threshold. Second, random point anomalies often occurred in satellite power subsystems. The occurrence of point anomalies was closely related to the health of satellite power subsystems and could be used to predict their life-span. The detection rate of point anomalies could be used to evaluate the model. Third, the model must be able to detect common satellite power subsystem contextual anomalies [8], such as main error amplifier (MEA) circuit failure and battery set open-circuit failure. The MEA value is larger than the normal value, which leads to lower discharge current when MEA circuit failure occurs. There are several sets of batteries on the satellite. When a group of batteries has an open-circuit failure, the battery set discharge current of the group becomes 0, and the discharge current of the other group of batteries increases.

## 4. Experiment and Discussion

### 4.1. Data Exploration and Preprocessing of the Telemetry Data

**Figure 4:**
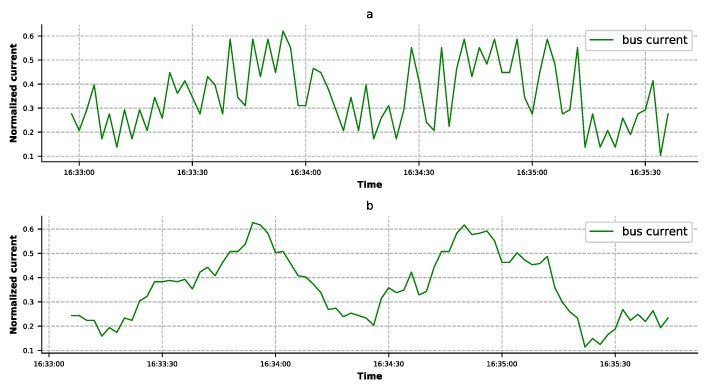
Comparison of the original bus current and the one processed by the moving-average method. (**a**) The original bus current. (**b**) The bus current processed by the moving-average method.

The methodology was applied to analyze the real telemetry data of a navigation satellite power subsystem. The original data set contained 65 features and 102,472 rows, and was acquired in December 2016 with a frequency of 2 s. We needed to select the appropriate features in the telemetry data to train the model. According to expert knowledge, the following kinds of features should be removed—(1) backup features, i.e., the backup values of other features; (2) low-frequency sampled features—the values that are the same as their corresponding high-frequency sampled features; (3) switch features—the switch value is unchanged; and (4) mark features—for example, battery charging overvoltage protection mark, which marks the serious faults in a satellite. At this time, the satellite's state could only be judged by this feature. After feature selection and removing the rows with excessive missing values, a data set with 34 features (as shown in Table 2) and 96,717 rows was generated. About 80,000 rows from these data sets were selected as the training data, and the rest as the test data. Among these features, the bus current required special handling (as shown in Figure 4a). The original value of the bus current fluctuated greatly, which the neural network treated as noise. In order to solve this problem, we could deal with this feature using the moving-average method [32], so as to eliminate the factors of accidental change and find out the development trends of the feature (as shown in Figure 4b).

The time-signal plots of features can help to find out the changing rule of each feature over time and the relationships between features. According to the observations and expert knowledge, the selected 34 features could be divided into 7 groups, each of which had features that monitored the same physical quantities, such as current and voltage, or did not affect each other during training. The grouping was as follows:

- Bus current and temperature;
- Battery set whole voltage and battery set single voltage;
- BDR/BCR output current and battery set discharge current;
- BEA and solar cell array current;
- BCR input current;
- Battery set charge current;
- MEA voltage.

### 4.2. Model Training

**Figure 5:**
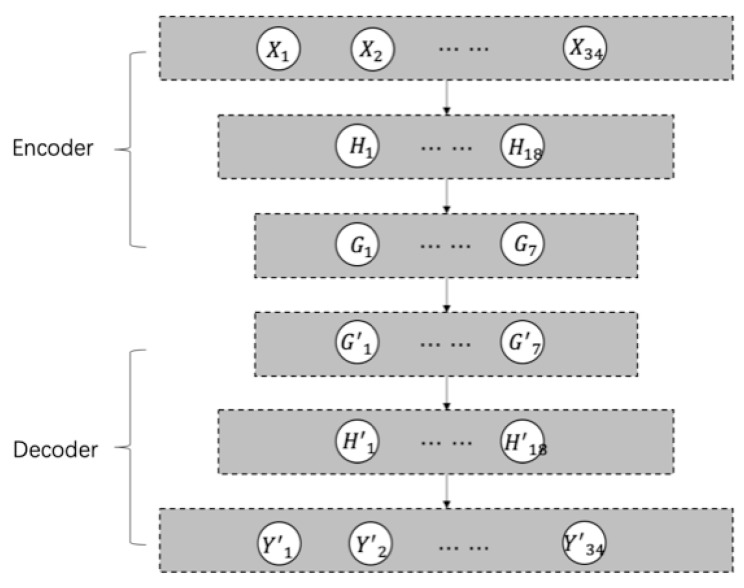
The architecture of the autoencoders.

**Figure 6:**
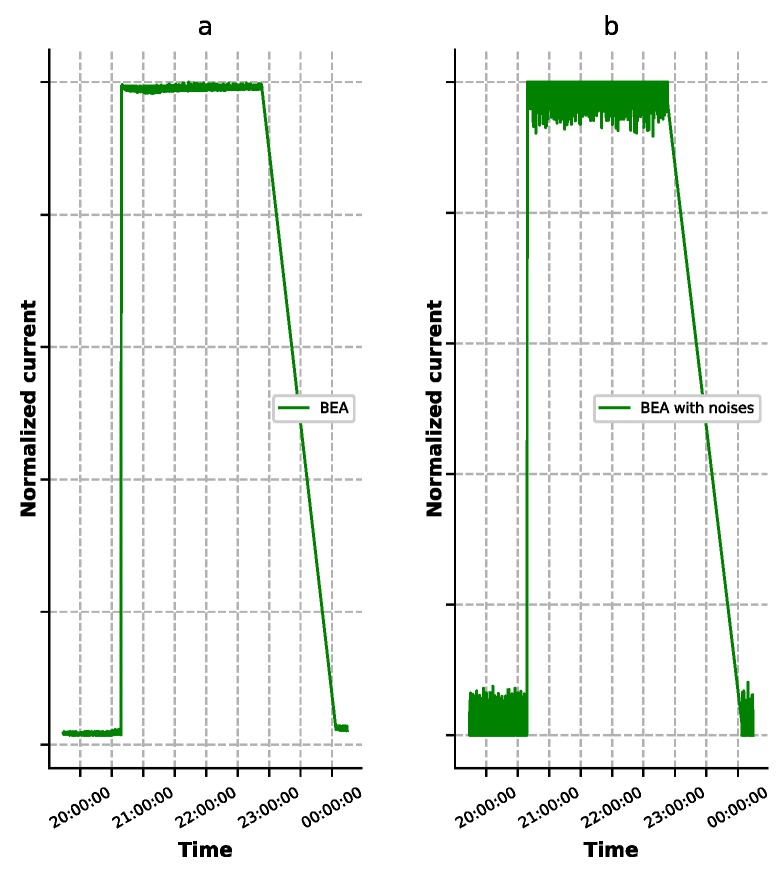
Comparison of the original BEA value with the BEA value with Gaussian noise. (**a**) The BEA value. (**b**) The BEA value with Gaussian noise.

In order to eliminate this big reconstruction error and make the autoencoder model more reliable, we used the method mentioned above to train the autoencoder in stages. The architecture of the four types of autoencoders is shown in Figure 5. Since the experimental dataset contained 34 features, the size of the input and output of the autoencoder was 34. As mentioned above, these 34 features could be divided into 7 groups, so the dimension of the middle layer was 7. The training of ‘ST-DAE’ was divided into 7 stages. Each training stage trained a set of features grouped above. The training order and the features of each stage were as follows—(1) BCR input current; (2) battery set charge current; (3) MEA voltage; (4) bus current and temperature; (5) battery set whole voltage and battery set single voltage; (6) BDR/BCR output current and battery set discharge current; and (7) BEA and solar cell array current. At each stage, the values of the untrained features were set to 0.5. The denoising autoencoder could be developed by adding additive isotropic Gaussian noise into the inputs. The comparison between the original value of BEA and the one with Gaussian noise is shown in Figure 6. In the experiment, the common activation functions were tested, and the Tanh function was finally selected. For the other hyperparameters, such as batch size and learning rate, Bayesian optimization was applied to search for the optimal values. The search ranges were [4, 8, 16, 32, 64, 128, 256] and [$10^{-4},10^{-3},10^{-2},\;10^{-1}$], respectively.

### 4.3. Performance Evaluation

This section evaluates the performance of the autoencoders from the following three perspectives, i.e., model reconstruction capability, contextual anomalies detection capability, and point anomalies detection capability.

#### 4.3.1. Evaluation on Model Reconstruction Capability

**Figure 7:**
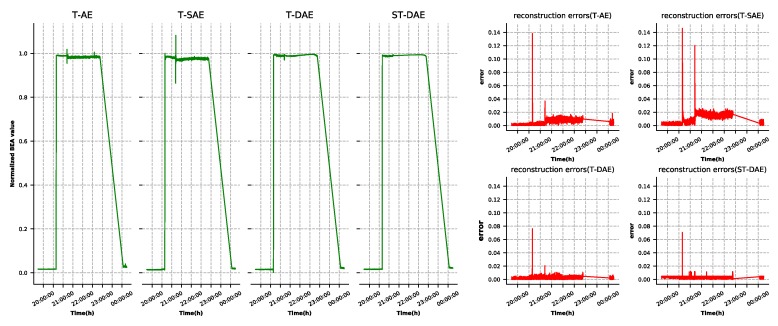
BEA reconstruction results and errors.

**Figure 8:**
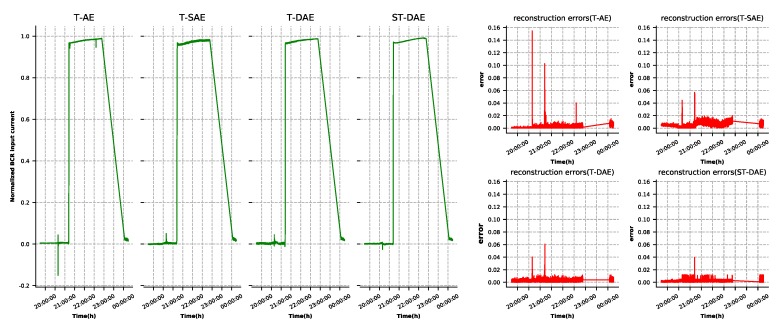
Battery charge regulator (BCR) input current reconstruction results and errors.

**Figure 9:**
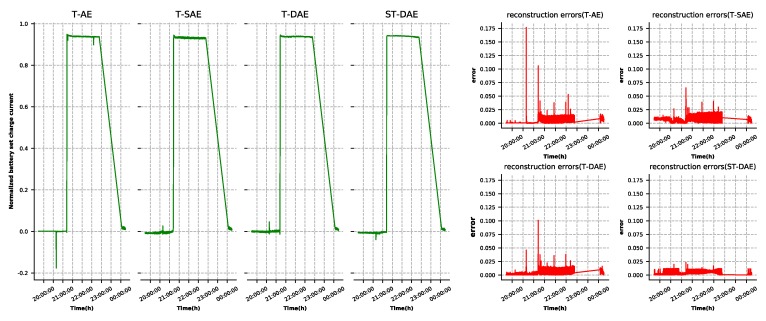
Battery set charge current reconstruction results and errors.

A well-developed autoencoder model must have the ability to reconstruct the input data. As shown in Figure 3, BEA, BCR input current, and battery set charge current had large reconstruction errors in some points due to the interaction between the features. This situation could lead to false alarms. The three features’ reconstruction results and errors generated by the four types of autoencoder, as described in Section 4.2, are shown in Figure 7, Figure 8 and Figure 9, respectively. It was observed that the denoising autoencoder had a better effect than the sparse autoencoder, in removing the interaction between features, and the stage-training method further enhanced the effect. The number of false alarms of each model could further prove this conclusion. According to the method mentioned in Section 3.3, we could observe that the normal cluster center was 0.046, the anomaly cluster center was 0.502, and the anomaly threshold was 0.275. Finally, the number of false alarms of each model was 776 (T-AE), 545 (T-SAE), 98 (T-DAE), and 8 (ST-DAE). The results indicated that stage-training denoising autoencoders (ST-DAE) were able to eliminate the false alarms, to a great extent.

#### 4.3.2. Evaluation on Point Anomalies Detection Capability

The occurrence of point anomalies is closely related to the health of satellite power subsystems. In order to verify the model’s point anomaly detection capability, 2,000 point noises which conform to the standard normal distribution were randomly added to the test dataset, which contained 34 features and 16,700 rows. The experimental results are shown in Table 3. Since our purpose was to detect anomalies, we needed to take the point anomalies as positive samples. Accuracy and recall rates were calculated in a method different from the usual and the calculation methods considered to be ‘correct’ in this study, were TN/(TN+FN) and TN/(TN+FP), respectively. The accuracy and recall of these four autoencoders were high. Their difference was mainly reflected in the recall rate. The recall rates from small to large were T-AE, T-SAE, T-DAE, and ST-DAE, which also confirmed the content described in Section 4.3.1. This experiment result showed that these four types of autoencoder models had high point anomaly detection capabilities; ST-DAE performed better.

#### 4.3.3. Evaluation on Contextual Anomalies Detection Capability

**Figure 10:**
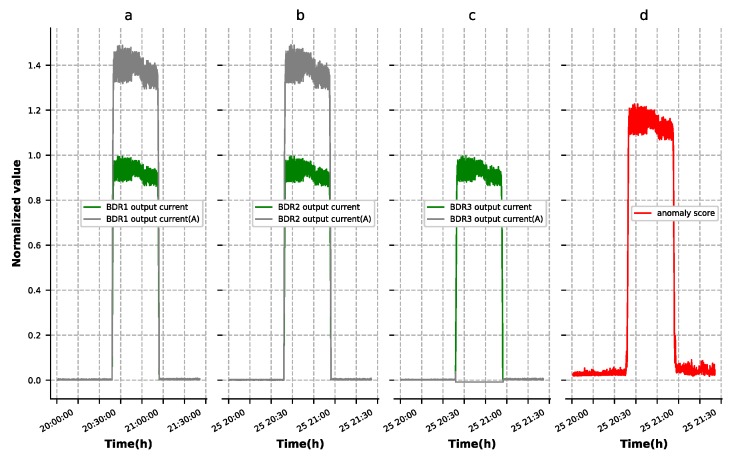
Detection result of the main error amplifier (MEA) circuit failure ((A) indicates the anomalous curve). (**a**) The normal BDR1 output current and anomalous BDR1 output current. (**b**) The normal BDR2 output current and anomalous BDR2 output current. (**c**) The normal BDR3 output current and anomalous BDR3 output current. (**d**) The anomaly score of the reconstruction results.

**Figure 11:**
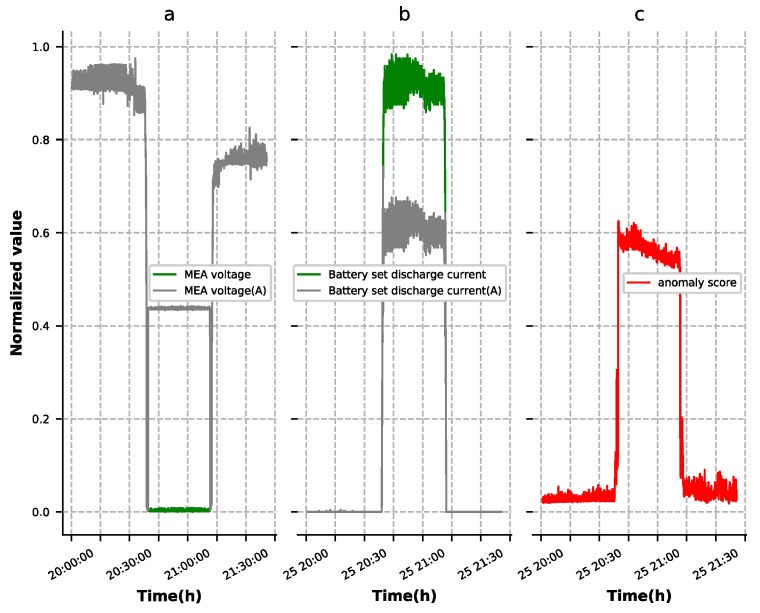
Detection result of the battery set open-circuit failure ((A) indicates the anomalous curve). (**a**) The normal MEA voltage and anomalous MEA voltage. (**b**) The normal battery set discharge current and anomalous battery set discharge current. (**c**) The anomaly score of the reconstruction results.

The contextual anomalies were determined within a specific context and usually reflected in several features. As shown in Figure 10, the third group of battery output current dropped to zero when this group of batteries had an open circuit fault. In order to maintain the power supply to the load, another two sets of battery power supply current increased. Since the autoencoder model we trained did not learn this data distribution, the anomaly scores as shown in Figure 10d increased greatly, during failure. Detection result of the battery set’s open-circuit failure is shown in Figure 11. The MEA voltage (A), as shown in Figure 11a, showed anomalous behavior where values were larger than normal ones, which led to battery set discharge current being smaller than normal, as shown in Figure 11b. Similarly, the anomaly scores, as shown in Figure 11c, increased greatly during failure, as shown in Figure 10d. Figure 10 and Figure 11 show the experimental results with ST-DAE as an example. In the process of experiment, we found that these four autoencoders had approximately the same detection effect for contextual anomalies. This showed that the autoencoder had strong contextual anomaly detection capability.

## 5. Conclusion and Future Work

Autoencoder is one of the most advanced techniques in unsupervised learning. This study proposed a new anomaly detection method for a satellite power subsystem based on state-training denoising autoencoder. In this work, based on the expert knowledge and full analysis of satellite telemetry data, the features of the data were trained in the denoising autoencoder model, in stages. A model trained in this manner has good reconstruction capabilities and can greatly limit false alarms. Four sets of comparative experiments were set up to illustrate the reliability of this method. In this study, a new cluster-based anomaly threshold determination method was proposed. The research results showed that the proposed state-training denoising autoencoders (ST-DAE) and threshold determination method could successfully identify contextual and point anomalies in satellite telemetry data.

In the process of research, we found that although autoencoders have a strong reconstruction ability, it is difficult to learn meaningful high-level features. It is difficult to achieve this purpose by adding constraints to the existing autoencoder models. In future, we intend to restrict the training of autoencoders by changing the initialization method of autoencoder weights and pruning the autoencoder to enable it to learn meaningful high-level features.

## Figures and Tables

**Table 1:** Description of the control experiments.

Autoencoders	Autoencoder Type	Training Method
T-AE	Common	Traditional
T-SAE	Sparse	Traditional
T-DAE	Denoising	Traditional
ST-DAE	Denoising	Stage-training

**Table 2:** Description of sensors.

Sensor Classes	Numbers
Bus current	1
Battery set charge current	1
Battery set discharge current	1
BCR input current	2
BDR /BCR output current	6
Temperature	4
Solar cell array current	2
Battery set whole voltage	2
Battery set single voltage	11
MEA voltage	2
BEA	2

**Table 3:** Point anomaly detection results.

Autoencoders.	True Positive	False Negative	False Positive	True Negative	Recall	Precision
T-AE	14591	109	186	1814	0.90700	0.94331
T-SAE	14611	89	191	1809	0.90450	0.95311
T-DAE	14690	10	63	1937	0.96850	0.99486
ST-DAE	14695	5	12	1988	0.99400	0.99749
